# Benchmarking signal quality and spatiotemporal distribution of interictal spikes in prolonged human iEEG recordings using CorTec wireless brain interchange

**DOI:** 10.1038/s41598-024-52487-5

**Published:** 2024-02-08

**Authors:** Amir Hossein Ayyoubi, Behrang Fazli Besheli, Michael M. Quach, Jay R. Gavvala, Alica M. Goldman, Chandra Prakash Swamy, Eleonora Bartoli, Daniel J. Curry, Sameer A. Sheth, David J. Francis, Nuri F. Ince

**Affiliations:** 1 Department of Biomedical Engineering, University of Houston, Houston, TX USA; 2 Department of Neurologic Surgery, Mayo Clinic, Rochester, MN USA; 3 Department of Neurology, Texas Children’s Hospital, Houston, TX USA; 4 Department of Neurology, UTHealth, Houston, TX USA; 5 Department of Neurology, Baylor College of Medicine, Houston, TX USA; 6 Department of Neurosurgery, Baylor College of Medicine, Houston, TX USA; 7 Department of Neurosurgery, Texas Children’s Hospital, Houston, TX USA; 8 Department of Psychology, University of Houston, Houston, TX USA

**Keywords:** Biomedical engineering, Translational research

## Abstract

Neuromodulation through implantable pulse generators (IPGs) represents an important treatment approach for neurological disorders. While the field has observed the success of state-of-the-art interventions, such as deep brain stimulation (DBS) or responsive neurostimulation (RNS), implantable systems face various technical challenges, including the restriction of recording from a limited number of brain sites, power management, and limited external access to the assessed neural data in a continuous fashion. To the best of our knowledge, for the first time in this study, we investigated the feasibility of recording human intracranial EEG (iEEG) using a benchtop version of the Brain Interchange (BIC) unit of CorTec, which is a portable, wireless, and externally powered implant with sensing and stimulation capabilities. We developed a MATLAB/SIMULINK-based rapid prototyping environment and a graphical user interface (GUI) to acquire and visualize the iEEG captured from all 32 channels of the BIC unit. We recorded prolonged iEEG (~ 24 h) from three human subjects with externalized depth leads using the BIC and commercially available clinical amplifiers simultaneously in the epilepsy monitoring unit (EMU). The iEEG signal quality of both streams was compared, and the results demonstrated a comparable power spectral density (PSD) in all the systems in the low-frequency band (< 80 Hz). However, notable differences were primarily observed above 100 Hz, where the clinical amplifiers were associated with lower noise floor (BIC-17 dB vs. clinical amplifiers <  − 25 dB). We employed an established spike detector to assess and compare the spike rates in each iEEG stream. We observed over 90% conformity between the spikes rates and their spatial distribution captured with BIC and clinical systems. Additionally, we quantified the packet loss characteristic in the iEEG signal during the wireless data transfer and conducted a series of simulations to compare the performance of different interpolation methods for recovering the missing packets in signals at different frequency bands. We noted that simple linear interpolation has the potential to recover the signal and reduce the noise floor with modest packet loss levels reaching up to 10%. Overall, our results indicate that while tethered clinical amplifiers exhibited noticeably better noise floor above 80 Hz, epileptic spikes can still be detected successfully in the iEEG recorded with the externally powered wireless BIC unit opening the road for future closed-loop neuromodulation applications with continuous access to brain activity.

## Introduction

Following the success of deep brain stimulation in various neurological disorders, including essential tremor (ET) and Parkinson's disease (PD)^1^, neuromodulation technology is constantly growing and serves as an effective solution to treat patients with neurological and psychiatric disorders^2–4^. Some of the applications include spinal cord stimulation (SCS) for managing chronic pain^5,6^, Vagus nerve stimulation (VNS) in epilepsy and treatment-resistant depression^7,8^, deep brain stimulation (DBS) in movement disorders and epilepsy^9,10^, and responsive neurostimulation (RNS)^11–13^ for the control of seizures in epilepsy^14^.

All these solutions require surgical implantation of electrodes and implantable pulse generators (IPGs) to deliver electrical stimulation to the target structure and also record neural activity for closed-loop neuromodulation^15^. However, implantable systems face various challenges, including covering limited regions in the brain, power management, and limited access to the assessed neural data in a continuous fashion. Continued progress in this area closely depends on our understanding of the underlying mechanisms of the disease and how the modulation occurs. In this scheme, a new hardware technology that can record neural activity at a higher sampling rate from multiple sites and stimulate various brain regions simultaneously through a wireless interface will likely promote additional applications of neuromodulation. The Brain Interchange (BIC) system of CorTec [CorTec GmbH, Freiburg, Germany]^16^ is a wireless and externally powered bio-signal amplifier that provides a unique opportunity to continuously record over extended periods, which helps to assess neural data with higher temporal and spatial resolution compared to other available devices^17^ in the chronic setting. In this study, to the best of our knowledge, for the first time, we investigated the feasibility of recording prolonged human iEEG from three subjects (~ 24 h for each subject) in the epilepsy monitoring unit (EMU) with the BIC and commercially available clinical amplifiers simultaneously.

We quantified the power spectra, noise characteristics, and performance of amplifiers in capturing the interictal epileptiform discharges (IEDs)^18^. We reported the packet loss rate and its characteristics during the wireless neural data transmission in each case. Moreover, we conducted comprehensive simulations to inspect the performance of different interpolation methods for recovering the missing packets in signals at different frequency bands. We anticipate our experiments and results can be used in future works as a benchmark and serve as a reference.

The remaining of this paper is organized as follows. In the next section, we describe BIC unit modules, recording setups, software platform, and established recording model. Section “Results” provides the study results and statistical evaluations, and the discussion is drawn in Sect. “Discussion”.

## Materials and methods

### Data acquisition

We recorded iEEG data from three patients (two pediatric and one adult) diagnosed with refractory epilepsy at Texas Children’s Hospital (TCH) and St. Luke's Medical Center of Baylor College of Medicine (BCM). This study was approved by the Institutional Review Boards (IRBs) of the University of Houston, BCM, and Mayo Clinic and all experiments and methods were performed in accordance with relevant guidelines and regulations. Furthermore, an informed consent was obtained from all participants and/or their legal guardians prior to incorporating their data into this study. All the recordings were obtained in the EMU with the BIC unit (device ID: ID0098) and the clinical amplifiers at a sampling frequency of 1 kHz and 2 kHz, respectively. Relevant medical annotations, including seizure onset zones (SOZ) and surgery outcomes, were provided by the clinical team at the affiliated institutes. Table 1 provides the demographic information of the subjects included in this study.

**Table 1:** The demographic information of the subjects.

Subject	Gender	Age	Clinical amplifier	Sampling rate (kHz)	ADC resolution	Recorded channels	Electrode type	Recorded channels with BIC	Recording time
*P1*	M	33	NK (JE-120)	2	24 Bit	124	Depth	32	24 h 36 min
*P2*	F	12	NQ	2	16 Bit	236	Depth	32	23 h 37 min
*P3*	F	19	NQ	2	16 Bit	254	Depth	32	24 h 17 min

For each subject, a prolonged iEEG was acquired where the signal was split into two streams and simultaneously recorded using the BIC and Natus Quantum (NQ—Natus Medical Incorporated, Wisconsin, USA)^19^ or Nihon Kohden (NK-JE-120-Nihon Kohden Corporation, Tokyo, Japan)^20^ amplifiers in the EMU. The clinical amplifiers in this study had a recording capability extending up to 256 channels, whereas the BIC unit's recording capacity was confined to 32 channels. Therefore, a subset of channels was selected to record by the BIC unit. In two of the cases (P2 and P3), we recorded the iEEG with the BIC unit from those electrodes that included the SOZ. In the other case (P1), we recorded iEEG from an active region with epileptiform spikes reported by the clinicians. The raw iEEG was converted to bipolar derivation for further processing, and the corrupted channels were visually identified and removed from the analysis for both systems.

### BIC unit's modules

The BIC unit is an implantable system with sensing and stimulation capabilities dedicated for promoting brain-computer interface and closed-loop neuromodulation research. As an externally powered implant, the BIC unit can continuously stream neural data to a nearby communication unit connected to a computer via USB. The computer controls the implant and other modules to record neural data and generate therapeutic electrical stimulation to the brain. In this study, we used a benchtop unit developed only for research studies without any implantation to reduce surgical risks. The BIC's specifications and recording capabilities are provided in Table 2.

**Table 2:** The BIC unit's recording capabilities and general properties.

System recording capabilities			
Recording channels	32	Sampling rate	1kHz
ADC resolution	16 Bits	Frequency band-pass	~ 2 to 325 Hz
Amplification gains	750× (57.5 dB), 375× (51.5 dB), 188× (45.5 dB), 95× (39.5dB)		
Max. input voltage	4–30 ${{\text{mV}}}_{{\text{pp}}}$ (dependent on the amplification gain)		
System general properties			
Implant dimensions (mm^3^)	67 × 38 × 7	Power supply	Wireless
Stimulation frequency range	Single pulse-200 Hz	Max. stimulation pulse current	6.12 mA
Stimulation pulse width range	10–2500 µs	Charge balancing counter pulse	4× pulse width
Stimulation pulse shape	Asymmetric rectangular and biphasic fashion		
Communication	Bi-directional, radio frequency (2.45 GHz), 5 Mbit/s		
Powering	Inductive 120–140 kHz Power regulated on implant demand		
BIC reference	Any electrode contacts or group of electrode contacts		

**Figure 1:**
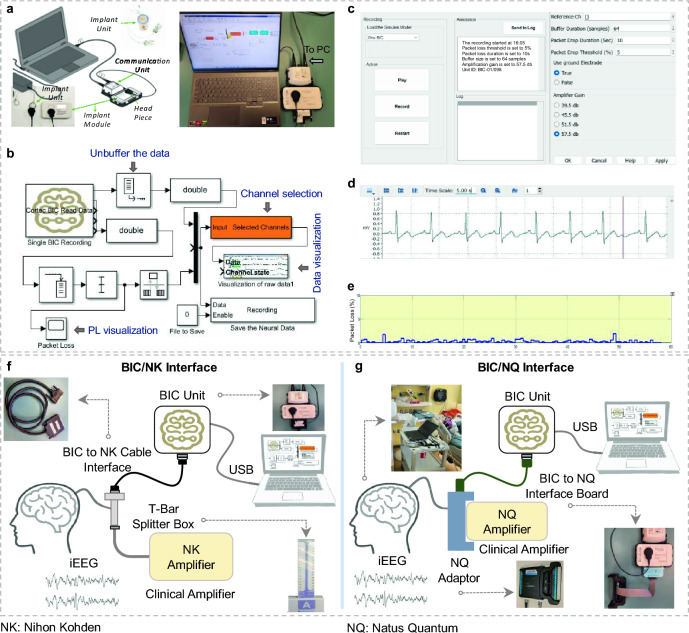
(**a**) The BIC unit modules (Implant module, head piece, and communication unit and its connection to the computing station). On the bottom left part of the figure, the implant module next to implant itself is depicted. (**b**) The SIMULINK model and data acquisition pipeline to record and visualize the neural data in real-time. (**c**) The GUI controls the recording properties such as PL threshold, buffer size, etc. (**d**) Real-time data visualization using g.HIsys toolbox in the SIMULINK model. A 5-s ECG signal was used for visualization purposes. (**e**) The panel shows the online level of PL for a 60-s recording segment. (**f**) Recording setup for the BIC system in each clinical site. The panel shows the setup at St. Luke's Hospital for the BIC and NK recording. The iEEG signal was divided into two streams using the splitter box. One stream goes to the clinical amplifier, and the other stream goes to the BIC unit using a custom-designed cable interface. (**g**) Shows the setup at the TCH for the BIC and NQ recording, where the signal was divided into two streams using the NQ adaptor and directed to the NQ amplifier and the BIC unit using a custom adaptor board.

As shown in Fig. 1a, the BIC benchtop unit consists of three main modules. 1. *The implant module,* which is plastic encased for the research phase and includes the implant, stimulation, wireless powering, and communication circuits. Along with these, the module contains electrical protection circuits to overcome the risk of an electrostatic discharge (ESD). The implant size is 67 × 38 × 7 mm^3^ and it has a maximum pulse repetition frequency of 200 Hz with maximum stimulation pulse amplitude of 6.12 mA. The maximum stimulation pulse width of the BIC is 2500 µs and the system delivers the stimulation in asymmetric rectangular and biphasic fashion. 2. *The head piece* produces an alternating magnetic field to wirelessly power the implant module. The energy transfers inductively in the range of 120–140 kHz. 3. *The communication unit* connects to the computer and powers the head piece. It is responsible for the wireless communication with the implant module. The communication unit operates using a proprietary protocol in the 2.4 GHz radio frequency band. The operational voltage of the communication unit is 5 V (USB) with typical power uptake between 2 and 3 W, depending on the system state and the distance between the implant and inductive transmission module. To maintain recording consistency, we placed the implant module, headpiece, and communication unit side by side within a 3D-printed frame. A brief comparison of the BIC and other implantable systems is provided in the Supplementary Table 1.

### Recording model and setup pipeline

We developed a SIMULINK model (Fig. 1b) implemented in MATLAB 2020b (MathWorks, Inc., Natick, MA, US) to acquire and visualize the neural data with the BIC system. Level-2 S functions were built based on the application programming interface (API, version 1.0.65) provided by CorTec, to communicate with the BIC kit and acquire data. The API offers users four different amplification gain factors to select from (see Table 2) ranging from 95× to 750×. Among these gain options, we selected the highest amplification gain (750×, 57.5 dB) for the iEEG recordings in this study to utilize the highest quantization resolution. A graphical user interface (GUI) was developed to control the SIMULINK model and the BIC unit (Fig. 1c). Moreover, the 32-channel streaming data was visualized in real-time using g.HIsys toolbox (g.Tec, Graz, Austria)^21^ for signal quality inspection. Figure 1d shows the real-time incoming stream of Lead -II ECG signal in a 5-s window recorded in the laboratory setting. The incoming data sampled at 1 kHz was buffered into 64 sample-long frames, and each transmission packet included data from all 32 channels.

The wireless transmission of neural data is associated with the risk of losing the data samples during the transfer process. This missing data, packet loss (PL), plays a significant role in determining the quality of the signal. The PL level might be affected by various factors, including the recording environment noise, communication unit location, and radio frequency collision^22^ caused by nearby devices. The data transmission channel is the frequency sub-band that is used by the BIC to wirelessly transfer the recorded data from the implant to the communication unit. A high level of PL, caused by interference in the transmission channel, results in poor signal quality or the loss of the content. Therefore, it is necessary to automatically switch the transmission channel to maintain good recording quality. To overcome the aforementioned problem, a controlling mechanism was added to the software. In the GUI the user can define an arbitrary threshold for the average PL, and the system looks for an alternative transmission channel in case the PL exceeds the specified threshold for a predefined period of time. The BIC system's available transmission channels are provided in Supplementary Fig. 1. In this study, the PL threshold for the recordings was set to 5% for a 10-s window to force transmission channel switching when PL is above the threshold for more than 10 s. The procedure for the selection of an alternative transmission channel is inherently stochastic. Nevertheless, given the availability of multiple channel options, the recording model will continue looking for an alternative band until it successfully adheres to the imposed constraints. The online PL-level visualization for a 60-s window of the recording is depicted in Fig. 1e.

In order to have a simultaneous recording with the BIC and clinical amplifiers (NK and NQ) without causing any interference to the clinical recording, two custom adapters were designed and fabricated to facilitate the process. The recording setup for each clinical site is depicted in Fig. 1f,g. In the BIC/NK configuration, the iEEG signal was divided into two distinct streams using a splitter box. One group was routed to the clinical system, while the other stream was directed to the BIC unit via our custom-designed cable interface. In the BIC/NQ setup, the iEEG stream was similarly divided using a custom 3D-printed NQ adaptor (Fig. 1g), which is manufactured by Ripple^23^. One stream was directed to the NQ system, and the other stream went to the BIC unit using our specially designed-adaptor boards.

### Signal processing

#### Amplifiers noise floor comparison and spectral analysis

The noise floor of the amplifiers was estimated by conducting shorted input tests. Specifically, to remove differential potentials between inputs and the reference, the input and reference channels of each amplifier were shorted and connected to the ground. A 1-min-long recording was obtained in this setup. The root mean square (RMS) values of the recorded signals were computed to characterize amplifier noise levels. Additionally, the power spectral densities (PSDs) of the recordings were computed to characterize the amplifier noise floor spectrum. The recorded signals were high pass filtered at 1 Hz in the preprocessing to remove the DC offset and compare the signals fairly among amplifiers with different high pass filter settings. The PSDs of noise floor and iEEG recordings were computed using the Welch periodogram with a 1s-long Hanning window and 50% overlap in each channel and then averaged across all channels.

#### Spike analysis

Interictal spikes, originating from the synchronous firing of hyperexcitable neurons, represent abnormal electrical activities that can be associated with epilepsy^24–26^. Investigation of the spikes' patterns can be used to identify the epileptogenic zone and further understanding of the pathophysiology of neurological disorders^27,28^. In clinical terms, an interictal spike refers to a distinctive, sharp transient event typically lasting from 20 to 70 ms and is easily distinguishable from the background activities^29^. We employed an established spike detector to assess the performance of the amplifiers^30^. The employed detector used the adaptive statistical distribution model of the signal envelope for the detection of IEDs. In order to quantify the temporal evolution of spiking activities, we divided the 24-h recording into 12-min-long segments (~ 120 segments in total), and the detector was applied to each 12-min segment of iEEG recorded by each amplifier. We reported the spike rate over the most active 12-min segments and across all segments in the 24-h recordings. The active segments were defined by selecting those with a spike rate higher than the threshold set by Otsu's method^31^. The structural similarity^32^ for the results of the 24-h spike detection was calculated using the following:

1 $$Structural\, Similarity= \frac{(2{{\varvec{\mu}}}_{{\varvec{x}}}{{\varvec{\mu}}}_{{\varvec{y}}}+\boldsymbol{ }{{\varvec{C}}}_{1})(2{{\varvec{\sigma}}}_{{\varvec{x}}{\varvec{y}}}+\boldsymbol{ }{{\varvec{C}}}_{2})}{({{\varvec{\mu}}}_{{\varvec{x}}}^{2}+{{\varvec{\mu}}}_{{\varvec{y}}}^{2}+\boldsymbol{ }{{\varvec{C}}}_{1})({{\varvec{\sigma}}}_{{\varvec{x}}}^{2}+{{\varvec{\sigma}}}_{{\varvec{y}}}^{2}+\boldsymbol{ }{{\varvec{C}}}_{2})}$$

where ${\mu }_{x}$, ${\mu }_{y}$, ${\sigma }_{x}$, ${\sigma }_{y}$ and ${\sigma }_{xy}$ are the mean values, standard deviations, and cross-covariance for each system and ${C}_{1}$ and ${C}_{2}$ are the regularization constants to avoid instability in the calculation if the mean or standard deviation is close to zero.

#### Packet loss analysis

Wireless transmission of neural data carries the risk of sample loss, resulting in degraded signal quality or, in more serious instances, complete loss of content if the missing samples cannot be recovered. To characterize the effect of PL, on the iEEG data, several simulations were executed. We applied synthetic PL (1%, 5%, and 10%) to the iEEG data recorded by NK and NQ systems (specifically, we randomly selected sample positions to mimic packet loss that can happen throughout the continuous recordings) and reconstructed the signal using the previous value replacement (current in-use method in the BIC unit). Since both clinical recording systems are tethered, the recorded data does not suffer from packet loss related artifacts. Using this experiment, we aimed to characterize the effect of PL on the iEEG PSD. Moreover, we conducted a simulation to examine the effect of the amplifier's sampling frequency on the recovery of missing packets. We introduced 10% PL to the clinical amplifiers (NK and NQ) recorded iEEG, sampled at 2 kHz, and reconstructed the signals. Next, following a low pass filtering step with 500 Hz corner frequency, we down-sampled the data to 1 kHz and repeated the experiment to compare the PSD of the reconstructed signals at each sampling frequency for each subject.

Furthermore, in order to thoroughly investigate the impact of different interpolation methods on the missing packet recovery, we applied 10% synthetic PL to the down-sampled (1 kHz) iEEG recorded by the clinical systems and compared the PSD of the reconstructed signals using the previous value replacement, linear interpolation, and spline interpolation methods.

## Results

### Raw signal quality

**Figure 2:**
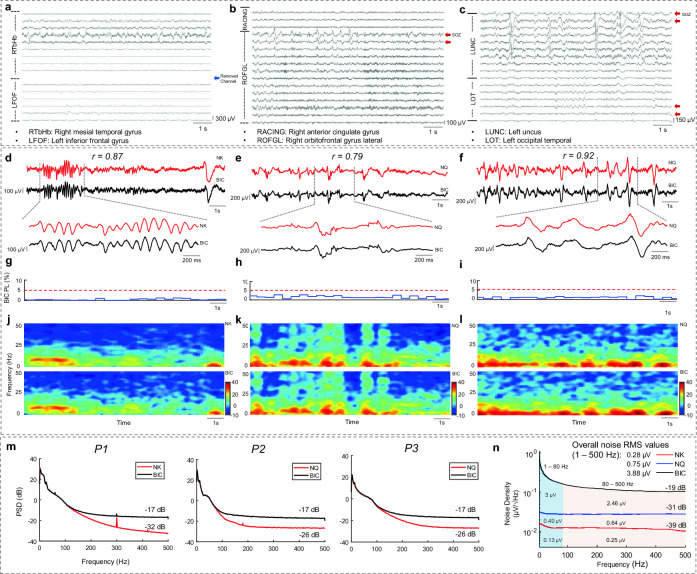
A 16-channel visualization of raw iEEG data recorded by the BIC unit for representative subjects (**a)** P1, (**b**) P2, and (**c**) P3. The removed noisy channel is marked with a blue arrow, and SOZ channels are marked using red arrows. (**d**) Raw data visualization for a 12-s segment of P1 (BIC and NK systems), (**e**) P2 (BIC and NQ systems), and (**f**) P3 (BIC and NQ systems), along with a 2-s zoomed signal for each group. The correlation coefficient of the visualized segments was 0.87, 0.79, and 0.92 for P1, P2, and P3, respectively. (**g**) BIC unit PL for the corresponding 12-s segment for P1, (**h**) P2, and (**i**) P3. The red dashed line indicates a 5% PL level. (**j**) Time–Frequency map for the selected 12-s window for P1, (**k**) P2, and (**l**) P3. (**m**) Power spectral density of the recordings. BIC system PSD reached − 17 dB, while the NK system reached − 32 dB at 500 Hz for P1. For P2 recording (BIC vs. NQ), the PSD for the BIC reached − 17 dB and − 26 dB for the NQ system at 500 Hz. We observed similar performance as P2 (− 17 dB for BIC and − 26 dB for NQ at 500 Hz) for the P3. (**n**) The noise floor of each system was estimated with the shorted input test. The overall noise RMS values, along with the low and high band RMS values, are reported for each band (e.g., the BIC system's noise floor below 80 Hz is 3.00 µV).

To provide a representative example of recorded signal quality and characteristics, 16-channel raw iEEG data recorded by the BIC for all three subjects are depicted in Fig. 2a–c. The corrupted channel 10 (right mesial temporal gyrus 13–14) in the P1 recording was removed in both NK and BIC recorded data. Furthermore, one channel of the raw iEEG for each system (BIC vs. Clinical Amplifier) is shown in Fig. 2d–f, along with the PL of the BIC unit for the corresponding segment in Fig. 2g–i. The time–frequency maps for the selected segments are provided in Fig. 2j–l for subjects P1, P2, and P3, respectively. The alpha-band oscillations (7–13 Hz) and a slow IED for P1, and multiple spiking activities for P2 and P3 were observed in the single-channel visualizations (Fig. 2d–f). The correlation coefficients for the visualized single channels were 0.87, 0.79, and 0.92 for P1, P2, and P3, respectively.

### Power spectral and noise floor

The average PSDs of the iEEG recordings were computed to assess the signal quality of the amplifiers and are shown in Fig. 2m. In all three cases, the iEEG PSD of the BIC reached a plateau above 200 Hz and settled around − 17 dB, likely approaching the noise floor of the amplifier. Consistently, the clinical amplifiers had PSD levels lower than BIC above 200Hz. The iEEG PSDs were almost identical, below 80Hz in all three cases. Figure 2n presents the results of the noise floor estimation and noise RMS values for each system in the frequency bands of 1–80 Hz and 80–500 Hz. The overall noise RMS values were 0.28, 0.75, and 3.88 µV for the NK, NQ, and BIC amplifiers, respectively, between 1 and 500 Hz. Both in the low and high band, the noise RMS values were smaller for the clinical amplifiers; 0.13, 0.40, and 3.00 µV, in the low band (1–80 Hz) and 0.25, 0.64, and 2.46 µV, in the high band (80–500 Hz), for the NK, NQ, and BIC systems, respectively. Although the BIC system had a noticeably higher floor than the clinical systems (see Fig. 2n), the iEEG PSDs below 80 Hz were almost identical, suggesting that the signal amplitude is much larger than the amplifier noise in the low band. Further details about the BIC unit noise characteristics, including the noise signal and it's PSD are provided in Supplementary Fig. 2.

### Spike analysis

**Figure 3:**
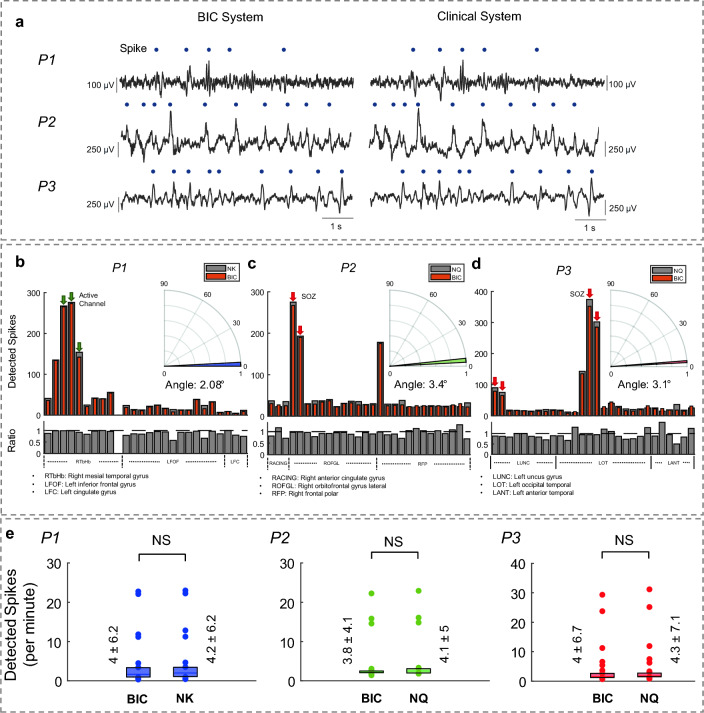
(**a**) Spike detection results over a 12-s window of the iEEG data for each subject. The detected interictal spikes are marked with blue circles. (**b**) A histogram of detected spikes over the recording segment with the highest spikes rate of each amplifier. Subject P1's results showed a ratio of 91% ± 7% between the BIC unit and NK system detected spikes rate. Clinically defined "active" channels are marked using green arrows. The bottom panel shows the ratio plot between the channel-by-channel number of detected spikes in each system. The gray dashed line, at 1, represents the complete alignment in both systems. To visualize the difference between the spike rates detected using the BIC and the NK system, we display displayed their angular distance in the inset (2.08°). (**c**) P2 results showed a 94% ± 14% ratio for BIC and NQ systems detected spikes. Clinically defined seizure onset zone (SOZ) channels are marked using red arrows. A 3.4° angle was observed for channel-by-channel comparison between BIC and NQ for this subject. (**d**) P3 results indicated the ratio of 93% ± 15% between BIC and NQ systems spikes rate, and the ratio plot is provided in the bottom panel. A 3.1° angle was observed in the channel-by-channel comparison for this subject between the systems. (**e**) Comparison of the number of detected spikes in each channel per minute by the BIC unit and clinical systems for each subject in the most active segment of the recordings. Statistical analysis results indicated no significant difference between the performance of BIC vs. NK and BIC vs. NQ in channel-by-channel spike detection.

The spike detection results for a 12-s iEEG data for each subject are shown in Fig. 3a. The spike rate distributions over channels in a 12-min-long recording segment, wherein the highest spike rate was observed for each amplifier, are presented in Fig. 3b–d top panels. The channel-by-channel ratio between the number of detected spikes in the BIC and the clinical systems streams (BIC/Clinical System) was calculated and shown in Fig. 3b–d bottom panels. In all three cases, we observed relatively high agreement where the average ratio was 91% ± 7%, 94% ± 14%, and 93% ± 15% for P1, P2, and P3, respectively. Additionally, we took the BIC and clinical amplifiers' channel-by-channel spike rates as vectors and estimated the angle between them. The angle between spike rate distributions of the systems was 2.08°, 3.4°, and 3.1° for P1, P2, and P3, respectively, within the selected most active segments. A non-parametric test (Kolmogorov–Smirnov) was used to statistically investigate the systems' performance in capturing the interictal spiking activities over each channel per minute in the selected segment (Fig. 3e). Our findings suggested no significant difference between the spike rates of BIC and clinical amplifier recordings (p-value: 0.67, 0.35, and 0.30 for P1, P2, and P3, respectively).

In Table 3, we further summarized the mean spikes rates per minute estimated over the 24-h analysis for each amplifier. The ratio of BIC spike rate vs. clinical amplifiers was 0.96, 0.95, and 0.95 for P1, P2, and P3, respectively.

**Table 3:** Average rate of detected interictal spikes over all channels per minute for the BIC unit and the clinical amplifiers during the 24-h recordings.

Subject	BIC spikes	Clinical system spikes	BIC/clinical system ratio	Number of channels (bipolar)
*P1*	33.6	35	0.96	26
*P2*	46.4	48.8	0.95	28
*P3*	59.2	62.1	0.95	28

**Figure 4:**
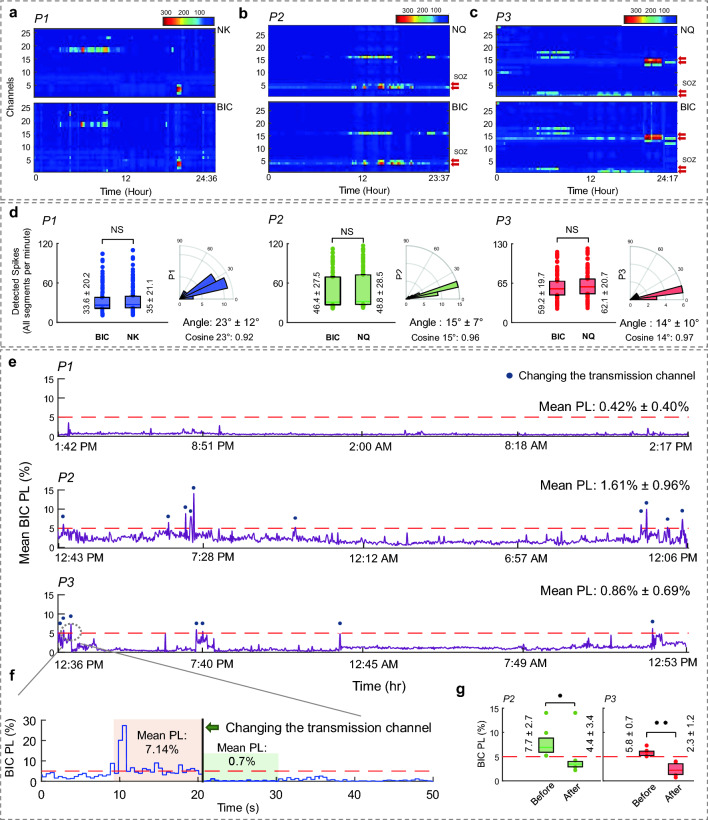
Spike detection results over the prolonged iEEG recording. The y-axis shows the channel numbers (Bipolar derivation), and the x-axis indicates the recording interval. (**a**) P1 spike results showed a 95.68% similarity between BIC and NK systems. (**b**) P2 results indicated a 98.13% similarity between BIC and NQ systems. (**c**) P3 results showed 95.64% similarity between BIC and NQ systems. Clinically reported SOZ channels are marked using red arrows. (**d**) Comparison of the overall number of detected spikes per minute with the BIC unit and clinical systems for each subject in all the recording segments. Statistical analysis results indicated no significant difference between the performance of BIC vs. NK and BIC vs. NQ in spike detection. The degree of similarity between the spike rate of the systems over the active segments in the prolonged recording is displayed as the angle on the right of each panel, showing 23° ± 12°, 15° ± 7° and 14° ± 10° angles between the systems in P1, P2, and P3 respectively. (**e**) The mean PL level for the BIC system for each subject is shown in the top three panels. The red dashed lines indicate a 5% PL level. Each point corresponds to averaged PL over a 10-s window. We observed 0.42% PL in P1, 1.61% in P2, and 0.86% in P3 recording. Time points when the transmission channel changed during the recordings are marked with blue circles in each plot. No switching happened during P1 recording; P2 and P3 had 10 and 7 transmission channel switches within 24 h of recording, respectively. A majority of the channel switches were observed during peak hours in the patients' rooms. Specifically in the early afternoon and early evening. (**f**) Shows the transmission channel switching process. The PL level dropped from 7.14 to 0.7% by changing the channel. (**g**) Statistical comparison of the PL rate before and after the transmission channel switch. A significant difference was observed between the results for P2 and P3, with p-values of 0.035 and 0.0015, respectively.

Figure 4a-c, in a 2D histogram format, represent the spike distribution over channels in 12-min windows across the 24-h period for each stream. Using (1), we computed the structural similarity of spike rate temporal distribution over channels between BIC and clinical amplifiers. We observed 95.68% structural similarity between BIC and NK amplifiers in P1 over 24 h and 36 min of data. For P2, the structural similarity was 98.13% between BIC and NQ amplifiers over 23 h and 37 min of data. Finally, for P3, 95.64% similarity was observed over 24 h and 17 min of recording. As shown in Fig. 4d, no statistically significant difference was observed in the overall number of detected spikes per minute between BIC and other clinical amplifiers in any of the three cases (Kolmogorov–Smirnov test, p-value: 0.75, 0.62, and 0.65 for P1, P2, and P3, respectively). To further assess the agreement between spike rate distributions over all channels, the angle over the active segments (selected by the Otsu thresholding method) in the prolonged recording was calculated (Fig. 4d). We observed 23° ± 12° angle with a median of 24° (cosine 24°: 0.91) in P1, 15° ± 7° with a median of 14.4° (cosine 14.4°: 0.96) in P2, and 14° ± 10° with a median of 10.4° (cosine 10.4°: 0.98) in P3. Overall results suggested that not only the spike rates but also their spatial distributions agreed between amplifiers.

### Packet loss analysis

#### Packet loss rate over the prolonged recordings

The average PL (over 10-s windows) of the BIC stream for the prolonged recording was calculated and illustrated in Fig. 4e for each subject. The average PL over 24-h was below 5% in each case. The mean PL was 0.42% ± 0.40%, 1.61% ± 0.96% and 0.86% ± 0.69% for P1, P2, and P3, respectively. The PL threshold for the recording was set to 5% to trigger channel switching for wireless data transmission. The system automatically switched the transmission channel after the average PL was above the defined threshold (5%) in a 10-s window. We observed a notable concentration of channel switches during peak hours in the patients' rooms, specifically in the early afternoon and early evening. The process of changing the transmission channel is depicted in the bottom panel of Fig. 4f. The average PL dropped from 7.4 to 0.7% in the new channel. While for P1, the system always remained in the same transmission channel due to the low PL, for P2 and P3, the BIC unit switched its channel 10 and 7 times, respectively. The blue circles pinpoint the instances of transmission channel switches that occurred during the recordings. The transition to the new transmission channel is inherently stochastic, and it typically results in a significant reduction in the PL rate, as shown in Fig. 4g (paired t-test, p-values: 0.035 and 0.0015 for P2 and P3, respectively). Nonetheless, there are certain instances when the chosen channel fails to reduce the PL rate, prompting the system to perform another transmission channel switch.

#### Packet loss analysis over iEEG data

Prior to the PL analysis over the iEEG data, we investigated the effect of PL over a set of synthetic sinusoidal signals with different amplitudes and frequencies. The results of the reconstructed signals PSD and noise RMS values are provided in Supplementary Fig. 3. As expected, due to the random nature of PL, the error PSDs were uniformly distributed across the frequency, and the signal amplitude and reconstruction error level were linearly related. However, we also noticed that the error level also scaled linearly with signal frequency. Higher frequency signals had higher levels of reconstruction error due to the lower correlation between consecutive samples. Consequently, the simulation results confirm that the PL can induce different levels of noise based on the recorded signal characteristic.

**Figure 5:**
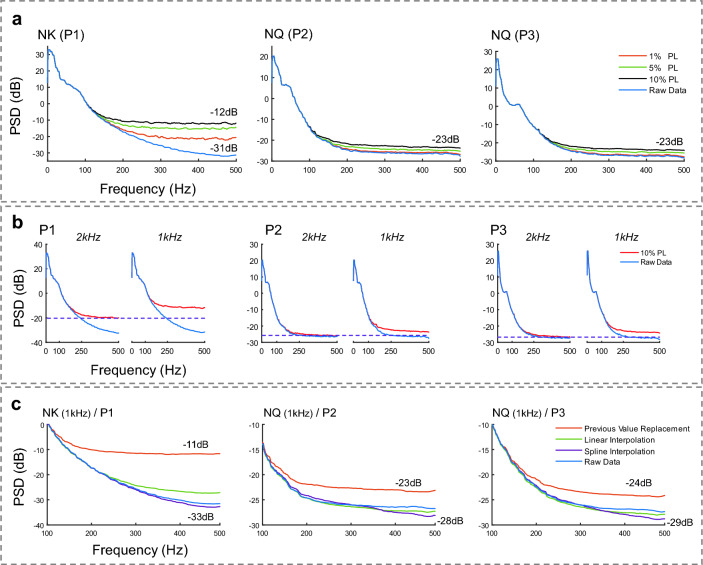
(**a**) Shows the effect of adding different synthetic PL levels (1, 5, and 10%) on the iEEG signal PSD for each subject (raw signal with no synthetic PL shown for comparison). The previous value replacement was used for signal reconstruction. (**b**) Shows the effect of the amplifier's sampling frequency in recovering the missing packets for synthetic 10% PL level. For each subject, the left plot shows the signal at the original sampling frequency (2 kHz) of the clinical systems, and the right plot shows the down-sampled signal to 1 kHz. (**c**) Effect of different interpolation methods on recovering the iEEG signal (down-sampled to 1 kHz) for each subject. The PSD of the reconstructed signal with the previous value replacement reached − 11 dB at 500 Hz, while the raw data PSD reached − 33 dB at 500 Hz for P1. For P2, the PSDs of the reconstructed signal with the previous value replacement and raw data were estimated at − 23 dB and − 28 dB, respectively, at 500 Hz. The previous value replacement reconstructed signal PSD reached − 24 dB while the raw data reached − 29 dB at 500 Hz in P3.

Figure 5a depicts the PSDs of the reconstructed iEEG signals with different PL levels for each subject. We introduced synthetic PL to a single-channel of iEEG signal obtained with the clinical systems to investigate the effect of the PL on the signal PSD at different frequency bands. Furthermore, to inspect the effect of sampling frequency on recovering the missing packets, we used the iEEG signal recorded by the clinical systems at 2 kHz and down-sampled to 1 kHz and added 10% of synthetic PL to the signals. We reconstructed the signals using the previous value replacement and presented the results in Fig. 5b. We observed an elevation in the PSD of the reconstructed signal with the 1 kHz sampling frequency compared to 2 kHz. The PSD raised from − 20 to − 11 dB, − 26 to − 23 dB, and − 27 to − 24 dB for P1, P2, and P3, respectively.

Figure 5c shows the simulation results to ascertain the optimal interpolation method exhibiting high accuracy and low computational complexity. We assessed the effect of three different interpolation methods on the signal quality by selecting a random iEEG segment recorded from the NK and NQ systems (down-sampled to 1 kHz) for each subject and adding 10% of synthetic PL to the data. Afterward, the interpolation methods (previous value replacement, linear interpolation, and spline interpolation) were used for signal reconstruction. For P1, we observed a substantial elevation in the reconstructed signal using previous value replacement compared to the raw iEEG signal after 100 Hz. The PSD of the reconstructed signal with the previous value replacement reached − 11 dB at 500 Hz, while the raw data PSD reached − 33 dB at 500 Hz. The PSD of the reconstructed signal using linear and spline interpolation methods overlapped with the raw data PSD below 250 Hz. However, notable differences emerged in the high band (> 250 Hz). Spline interpolation's smoothing characteristic and removal of high-frequency noise components resulted in subsidence in the PSD of the reconstructed signal compared to the raw data PSD above 350 Hz. For P2, the PSDs of the reconstructed signal with the previous value replacement and raw data were estimated at − 23 dB and − 28 dB, respectively, at 500 Hz. Furthermore, both linear and spline interpolations exhibited comparable performance to the raw data below 350 Hz. The previous value replacement reconstructed signal PSD reached − 24 dB while the raw data reached − 29 dB at 500 Hz in P3. Additionally, both linear and spline interpolations exhibited similar performance as observed in P2.

## Discussion

### Recording setup and amplifiers signal characteristics

In this study, we investigated the early feasibility of recording human neural data (iEEG) using the BIC unit, a wireless, portable, and externally powered bio-amplifier. We established a MATLAB/SIMULINK-based environment to simultaneously record prolonged iEEG with the BIC and clinical amplifiers (Natus Quantum or Nihon Kohden) from three subjects with refractory epilepsy. We estimated the PSDs of iEEG streams for all subjects. The results showed quite overlapping spectral characteristics between each pair (BIC vs. NK and BIC vs. NQ) for the recordings in the low band (< 80 Hz), and the primary distinctions were predominantly observed above 80 Hz. Specifically, above 200 Hz, the iEEG PSDs, regardless of the amplifier used, reached a plateau likely defined by the background noise level of the neural activity and the noise floor of the amplifier. Clinical tethered amplifiers generally had lower power levels (− 32 dB and − 26 dB for NK and NQ, respectively), while for the wireless BIC unit, the PSD reached − 17 dB. We estimated the noise floor of each system by conducting shorted input tests and reported the noise RMS values and findings correlated with the iEEG PSDs. Our results confirmed that the clinical amplifiers had lower noise RMS values (lower noise floor) in both low and high-frequency bands. While it was almost flat for the clinical amplifiers, the BIC amplifier noise spectrum followed the 1/f flicker noise characteristic^33–35^. We also observed localized "popcorn type" of sharp spiking noise (See Supplementary Materials section for sample raw data representing the noise characteristic). Additionally, the difference between the PSD of the recorded iEEG of each system and the amplifiers' noise floor is ascribed to the intrinsic noise content present in the neural signals.

### Performance over spike analysis

We assessed the BIC system's capability to capture clinically-relevant events by examining interictal spike detection. We performed interictal spike analysis over a 12-min-long segment of iEEG data associated with the highest spike rate for each subject. We provided the spike distribution over channels in Fig. 3b–d for all amplifiers, which showed a concordance of over 90% between the amplifiers. We compared our findings with the reported SOZ by the clinicians. The SOZ was included in the recorded 32 channels with the BIC for subjects P2 and P3. Since SOZ information was not available for P1, we recorded from an active region with interictal spikes. The clinically-reported active channels in P1 had the highest spike rate in both NK and BIC systems. Moreover, the clinically marked SOZ channels for P2 was congruent with those channels with the highest spike rate. For P3, two of the SOZ channels had the highest spike rates, and two were not comparably active in the analyzed segment (In both BIC and NQ systems). We took the spike distribution over all channels of each system as vectors and estimated the angle between them as a degree of similarity in the most active 12-min-long segment of the recordings. In each subject, the angles were measured below 3.5°, which indicates a high level of alignment between the detected spike distributions by the amplifiers in each channel. This agreement was supported by the statistical analysis, which indicated no significant distance between the spikes distributions in the BIC and clinical systems.

The spike detection results over 24-h recordings for all segments showed above 95% of structural similarity between BIC and clinical amplifiers. The statistical tests indicated no significant difference between the overall number of detected spikes across each segment for the amplifiers. A higher spike rate was evident in the clinical amplifiers compared to the BIC unit across all three subjects. The average spike rates per minute over all channels during the 24-h recording for the BIC vs. clinical systems were reported, and we observed an above 0.95 ratio between the rates for each subject. Between spike rate distributions over channels in the most active segments, selected using Otsu's thresholding method, of BIC and clinical amplifiers, we observed an angle below 24° (cosine 24°: 0.91) for each subject. As previously discussed, the iEEG PSDs showed a similar characteristic in the low band (< 80 Hz). Furthermore, the main energy of spiking activities is generally below 80 Hz. Therefore, this high level of agreement between the systems' spikes rates over channels and across the prolonged recordings was expected. Moreover, the BIC system had a higher amplifier noise floor between 1 and 80 Hz (Fig. 2n) which is likely masked by the iEEG signal power as shown in Fig. 2m, resulting in a greater overlap in the low band between the systems. In addition, as shown in Supplementary Fig. 3a,b error panel, although the packet loss has a uniform noise distribution over the spectrum, it affects the signal quality in the high band more than the low band due to the higher correlation between the samples in the low band. We anticipate that both the packet loss and the amplifier noise with broad band characteristics are contributors to the 4–5% difference in the detected interictal spike rate between the systems. Furthermore, we observed a higher concordance between the BIC and the clinical amplifiers for the prolonged data analysis, given that the recording channels were not consistently active throughout the 24-h recording, and the environmental noise may have varied over time (different levels of PL during the recording for each segment), thus not corrupting the data adversely. Consequently, we reported similarity rates for each subject that spanned the entire 24-h period rather than being based on a single selected segment. Our results validated the ability of the BIC system to reliably detect interictal spikes with a high degree of similarity with respect to the systems routinely used in clinical settings.

### Packet loss characteristics and signal reconstruction

The wireless transmission of neural data poses the threat of losing data samples resulting in poor signal quality or even losing the content. Therefore, an essential factor in the BIC signal quality is the PL level. Additionally, the 2.4 GHz frequency band (reserved for Industry, Scientific, and Medical (ISM) purposes) used by the BIC for data transfer overlaps with other wireless technologies such as Wi-Fi or Bluetooth. The presence of any nearby device using a frequency channel that overlaps with the in-use BIC system's transmission channel can interfere with the data transfer process. Nonetheless, multiple available transmission channels allow the system to switch to unoccupied channels, to maintain a low packet loss level. We inspected the effect of PL on signals with different amplitudes and frequencies. We observed that PL induces more severe distortion resulting in higher noise levels (more elevation in the PSD) in the synthetic signals at high-frequency bands due to the faster changes in the signal morphology. Moreover, our results indicated that the PL has more effects on the signals with higher amplitude due to the higher variance in the sample points. We then investigated the effect of PL on the iEEG signal PSD for each subject. The findings revealed a positive correlation between the PL level and the signal PSD. As we discussed, the PL has a greater impact on the noise level and power of the signals with higher amplitudes and frequencies. Hence, we observed a substantial elevation in the reconstructed signals PSDs for different synthetic PL levels in P1, attributed to the signal's amplitude and frequency content, compared to other subjects.

We examined the impact of the amplifier's sampling frequency on recovering the missing packets. An elevation in the PSD for the reconstructed signal at 1 kHz compared to the 2 kHz sampled signal was observed. The results pointed out a higher chance of accurately recovering the missing packets can be achieved in the signals sampled at higher rates due to the higher correlation between the samples. We studied the effect of interpolation methods on the signal quality. The current in-use method employed for interpolation in the BIC unit (previous value replacement) with linear and spline interpolations were compared. Our findings suggested a considerable improvement in the signal quality can be obtained (maintaining low computational complexity) by employing linear interpolation in the high-frequency bands, while all methods performed well in the low band (< 100 Hz). Furthermore, taking the rate of changes in signal morphology in the low-frequency band, having a PL threshold below 5%, is considered acceptable. However, a threshold of below 2% for PL in high-frequency band investigations is recommended.

### Final verdicts and future prospects

The goal of the study was to evaluate whether the BIC system is technically suitable for implantation in later studies for the iEEG acquisition and identification of IEDs in patients with epilepsy. Therefore, a performance comparison between the wireless benchtop version of the BIC system and tethered clinical amplifiers was carried out, during which we exposed three patients to our experimental setup as described above. The technical systems linked to the patients' externalized DBS leads posed no electrical risks and adverse events.

One limitation of this study is focusing mainly on the clinically-relevant events that have their main energy in relatively low-frequency bands (below 80 Hz, interictal spikes). The BIC system noise floor and signal characteristics were robust in the lower frequencies; thus, the system detected clinically-relevant events with a high degree of agreement with respect to the clinical systems. The recent advances in neural data processing^36–38^ and noise elimination^39–43^ are of importance for the next phase of this study. Furthermore, the achievements in the implantable devices developments have shown promising results in neuromodulation and neuro rehabilitation fields^44–47^. Future studies may benefit from the investigation of the high-frequency bands and detection of the high-frequency oscillations (HFO), specifically 80–500 Hz, where we can find HFOs as biomarkers for SOZ^48–51^. The statistical properties of the signal background activity (such as the standard deviation) are widely used in the amplitude threshold based HFO detectors^52,53^. The higher noise floor of the BIC could pose a potential challenge for the detection of high frequency oscillations as these events might get buried in the background activities and amplifier noise. To be more specific, the typical amplitude threshold which is 3–5 times of background standard deviation that is used in established detectors tuned for the clinical amplifiers could be too high due to the higher noise floor of the BIC and needs to be adjusted accordingly^42^. For this purpose, a larger subject pool and precise sampling of SOZ is required for the future work.

To conclude, we established a rapid prototyping and recording environment in MATLAB/SIMULINK for the BIC unit and evaluated it in the prolonged iEEG recordings over three human subjects. We acquired iEEG data with the BIC module, exhibiting below 2% average PL over the 24 h of recording for each subject, simultaneously with the clinical amplifiers. It is important to note that the reported PL rates for the recordings are likely an underestimation of the PL in a practical setting, as the head piece and communication unit were placed side by side in a 3D-printed frame. In this ideal scenario, we were already able to observe a difference in the PL rate for our recordings depending on the background noise levels of the hospital room environment. In scenarios where the BIC is implanted, higher PL rates would be anticipated. Lastly, despite the advantage of the tethered clinical amplifiers, showcasing a significantly better noise floor (< 25 dB), the BIC unit demonstrated its capability to record data of comparable quality, specifically in the low-frequency band (< 80 Hz), in a wireless fashion. This was substantiated by our spike analysis results, which concurred with the findings from the clinical systems and the SOZ channels reported by the clinician.

### Supplementary Information

Supplementary Information.

## Data Availability

The data that support the findings of this study will be openly available at the following URL/DOI: 10.18120/6szn-h592.
