# Supplementary material for: Benchmarking signal quality and spatiotemporal distribution of interictal spikes in prolonged human iEEG recordings using CorTec wireless brain interchange
Source: Sci Rep. 2024 Feb 8;14:2652. doi: 10.1038/s41598-024-52487-5 (PMC10853182; doi:10.1038/s41598-024-52487-5)
Supplement: Supplementary file 1 — Supplementary Information. [file 41598_2024_52487_MOESM1_ESM.docx]

***Supplementary Materials***

*Comparison of the BIC with other implantable systems:*

| System | Recording | Sampling Rate | No. Channels | Stimulation | Max. Stimulation Frequency | Max. Stimulation Current | Max. Pulse Width |
| --- | --- | --- | --- | --- | --- | --- | --- |
| Percept | ✓ | 250Hz | 16 (2×8) | ✓ | 250Hz | 25.5mA | 450 µs |
| Infinity | × | N/A | 16 (2×8) | ✓ | 240Hz | 12.75mA | 500µs |
| Vercise | × | N/A | 16 (2×8) | ✓ | 250Hz | 12.7mA | 450µs |
| RNS-320 | ✓ | 250Hz | 8 (2×4) | ✓ | 333Hz | 10mA | 1000µs |
| WIMAGINE | ✓ | 32Ch (1KHz)  64Ch (600Hz) | 64 | × | N/A | N/A | N/A |
| BIC | ✓ | 1000Hz | 32 | ✓ | 200Hz | 6.12mA | 2500µs |

**Supplementary Table 1**

The comparison of the BIC system with Medtronic Percept, Abbott Infinity, and Boston Scientific Vercise, DBS systems, Neuro pace RNS system and WIMAGINE (Wireless Implantable Multi-Channel Acquisition system for Generic Interface with NEurons) device which is for ECoG recording.

*BIC available Transmission channels:*


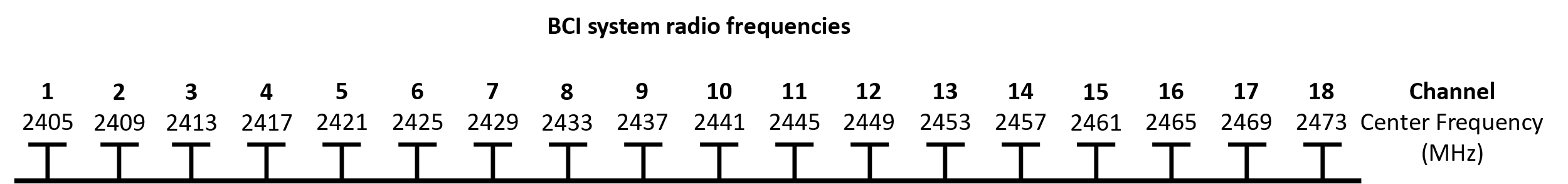


**Supplementary Figure 1**

BIC system’s available transmission channels. The BIC unit uses the 2.4GHz frequency band for data transfer process. The recording model will automatically change the transmission channel if the averaged level of packet loss goes beyond the predefined threshold by user.

The use of radio frequency bands is regulated in the USA, Europe, and other countries. The BIC system requires a high bandwidth which is achieved using a frequency band reserved for Industry, Scientific, and Medical (ISM) purposes in the range between 2.4 and 2.5 GHz. However, this band is also widely used by other wireless technologies such as Wi-Fi, Bluetooth, and Zigbee/IEEE 802.15.4 wireless data networks. A conflict between multiple devices using the same band is managed by dividing the band into several channels. The presence of any nearby device using a frequency band that overlaps with the in-use BIC system's transmission channel can interfere with the data transfer process. Nevertheless, given the availability of multiple channel options, the recording model will continue looking for an alternative channel until it successfully adheres to the imposed predefined (by the user) constraints.

*BIC Noise Signal and Noise Floor:*


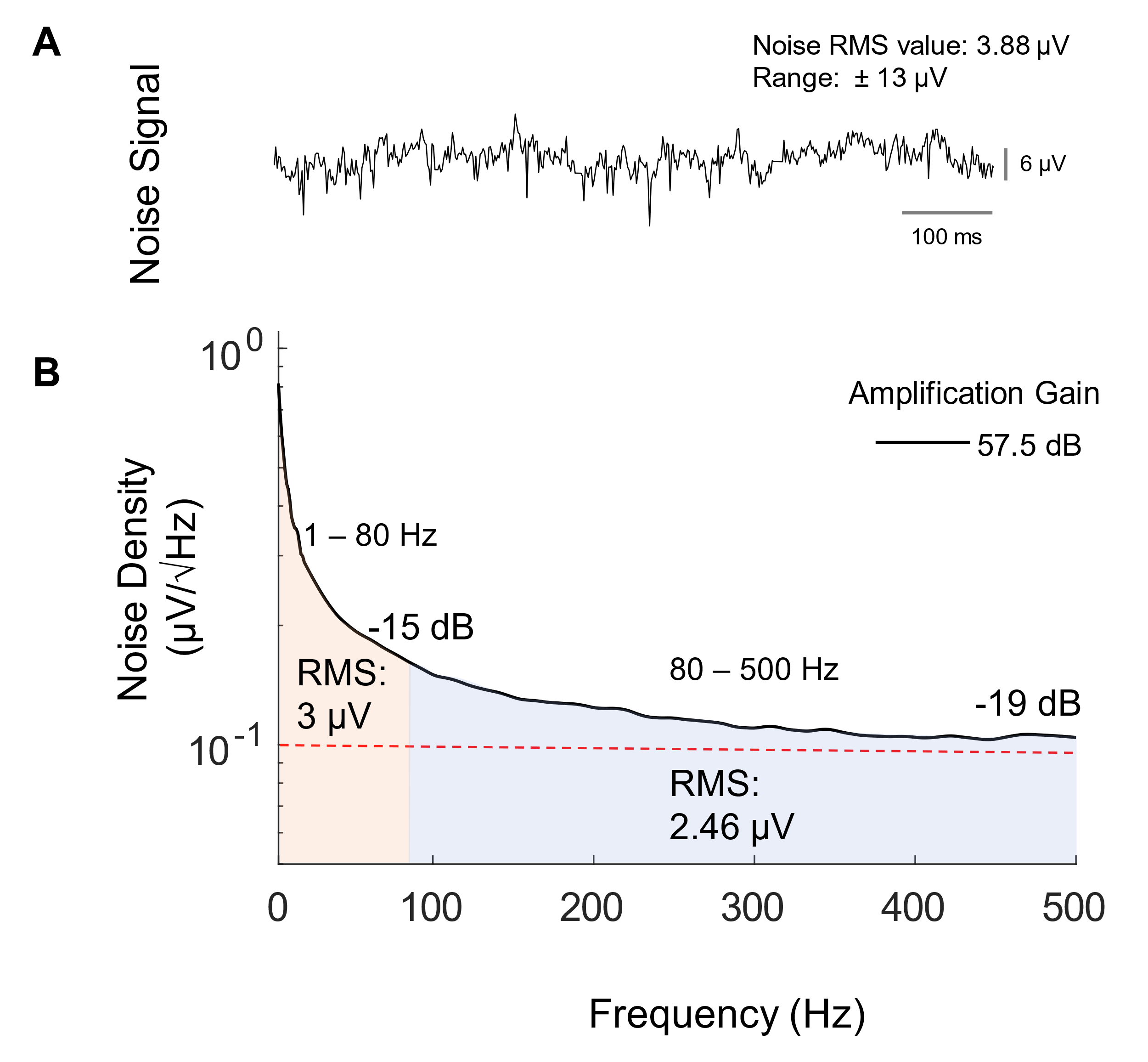


**Supplementary Figure 2**

**(A)** Visualization of the BIC system recorded noise (with shorted input test) for the 57.5 dB amplification gain. While fluctuations are inherent characteristics of noise, the presence of sharp transient artifacts (sparse popcorn noise in the form of large spikes) was observed in the noise which contributed to a dramatic noise floor elevation. **(B)** The shorted input test results indicated that for 57.5 dB amplification gain, the noise floor reached -15 dB at 80 Hz and -19 dB at 500 Hz.

*Packet Loss Analysis over Synthetic Data:*


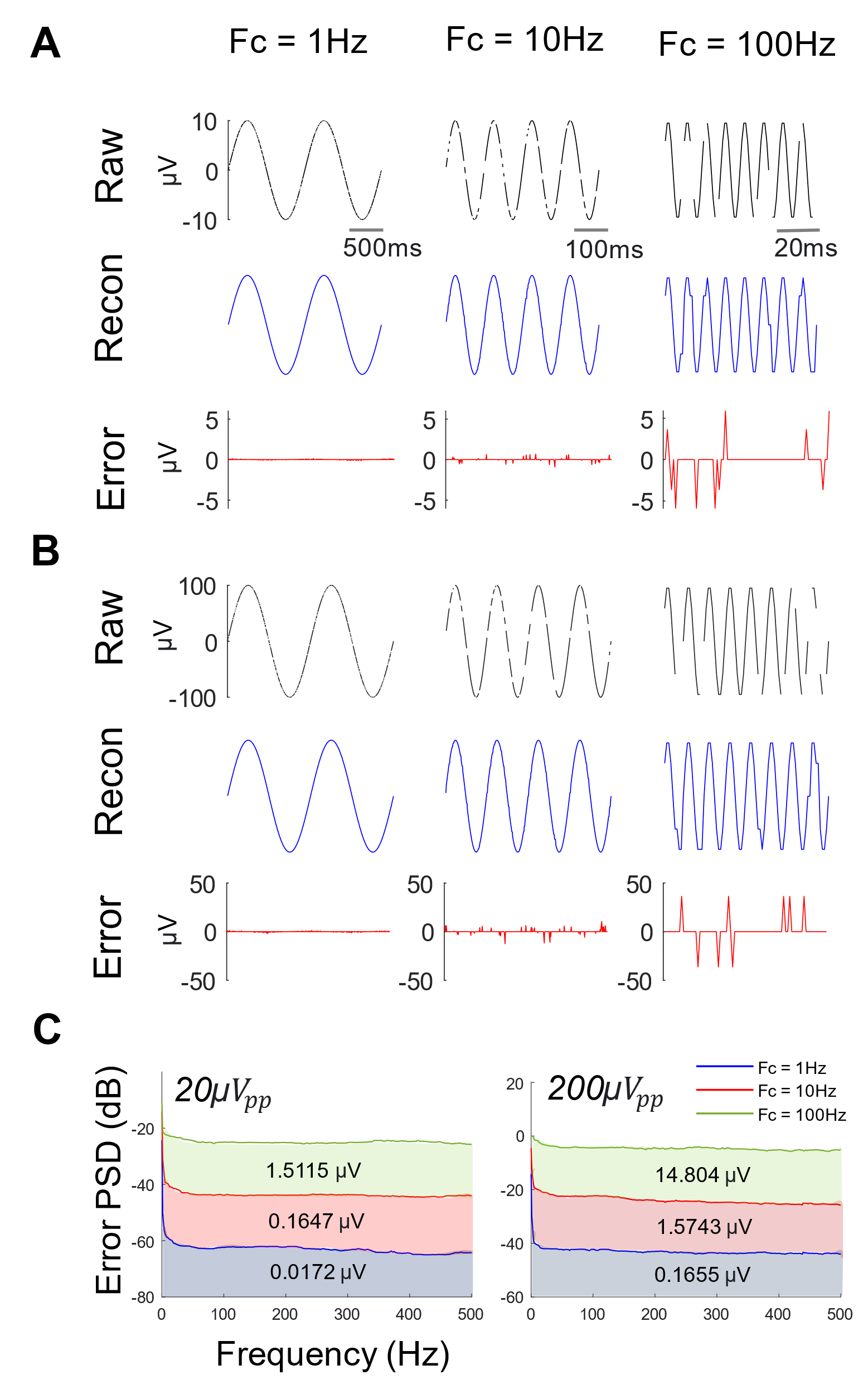


**Supplementary Figure 3**

Effect of 10% PL on the synthetic sinusoidal signals at different frequencies and amplitudes. **(A)** Shows the raw data with PL at 1 Hz, 10 Hz, and 100 Hz on the top panel, reconstructed signal using previous value replacement in the middle panel, and error (residual) values in the bottom panel for a 20 µ$V_{pp}$ signal. **(B)** Shows a similar concept for 200 µ$V_{pp}$ sinusoidal signal. **(C)** The PSD of the residual value (error) for each signal amplitude and frequency with the corresponding RMS values between 1 - 500 Hz. We observed 0.017 µV, 0.16 µV, and 1.51 µV for the error RMS values for 20 µ$V_{pp}$ signal and 0.16 µV, 1.57 µV, and 14.8 µV for 200 µ$V_{pp}$ at 1 Hz, 10 Hz, and 100 Hz sinusoidal signal, respectively, between 1 - 500 Hz.

To characterize the effect of PL, several simulations on synthetic signals were executed. First, a set of synthetic single-tone sinusoidal signals (1 Hz, 10 Hz, and 100 Hz) with 20 µ$V_{pp}$ (±10 µV range) and 200 µ$V_{pp}$ (±100 µV range) amplitude was defined to investigate the effect of PL on signal quality at various amplitudes and frequencies. We introduced 10% PL to the data and reconstructed the signals using the previous value replacement (current in-use method in the BIC unit). We computed the error (residual) values by subtracting the raw signals from the reconstructed and compared their PSDs and RMS values. These results suggest that when the missing packets are replaced with the previously available values, the introduced noise in the reconstructed signal scales with the signal amplitude but also with its frequency. While the low frequency signals are more correlated temporarily due to slow changes in signal amplitude, the correlation between neighboring signals reduces when the signal changes rapidly as in the case of a 100 Hz sinusoid. The low correlation between samples induced large artifacts when the missing data points are replaced with the available sample points proceeding them. Consequently, PL could induce higher level of broad band noise depending on the frequency content of the signal being recorded.
